# Gene signature characteristic of elevated stromal infiltration and activation is associated with increased risk of hematogenous and lymphatic metastasis in serous ovarian cancer

**DOI:** 10.1186/s12885-019-6470-y

**Published:** 2019-12-30

**Authors:** Huiran Yue, Jieyu Wang, Ruifang Chen, Xiaoman Hou, Jun Li, Xin Lu

**Affiliations:** 1 Obstetrics and Gynecology Hospital, Fudan University, No.419, Fangxie Road, Shanghai, 200011 People’s Republic of China; 2 Shanghai Key Laboratory of Female Reproductive Endocrine Related Diseases, Shanghai, 200011 China

**Keywords:** Hematogenous and lymphatic metastasis, Lymphovascular space invasion, Tumor stroma, Cancer-associated fibroblast, Ovarian cancer

## Abstract

**Background:**

The clinical significance of hematogenous and lymphatic metastasis in ovarian cancer has been increasingly addressed, as it plays an imperative role in the formation of both intraperitoneal and distant metastases. Our objective is to identify the key molecules and biological processes potentially related to this relatively novel metastatic route in serous ovarian cancer.

**Methods:**

Since lymphovascular space invasion (LVSI) is considered as the first step of hematogenous and lymphatic dissemination, we developed a gene signature mainly based on the transcriptome profiles with available information on LVSI status in the Cancer Genome Atlas (TCGA) dataset. We then explored the underlying biological rationale and prognostic value of the identified gene signature using multiple public databases.

**Results:**

We observe that primary tumors with increased risk of hematogenous and lymphatic metastasis highly express a panel of genes, namely POSTN, LUM, THBS2, COL3A1, COL5A1, COL5A2, FAP1 and FBN1. The identified geneset is characterized by enhanced deposition of extracellular matrix and extensive stromal activation. Mechanistically, both the recruitment and the activation of stromal cells, especially fibroblasts, are closely associated with lymphovascular metastasis. Survival analysis further reveals that the elevated expression of the identified genes correlates to cancer progression and poor prognosis in patients with serous ovarian cancer.

**Conclusions:**

Our findings indicate that tumor stroma supports the hematogenous and lymphatic spread of ovarian cancer, increasing tumor invasiveness and ultimately resulting in worse survival. Thus stroma-targeted therapies may improve the clinical outcomes in combination with cytoreductive surgery and chemotherapy.

## Background

Ovarian cancer is the most lethal gynecological malignancy, and a fair number of patients are diagnosed in advanced stage with extensive intraperitoneal spread and distant metastases [1]. Explorations regarding the underlying mechanisms of ovarian cancer metastasis thus contribute to the development of targeted therapies, improving the clinical outcomes in patients with this life-threatening disease.

It has long been assumed that direct shedding of ovarian cancer cells from the primary site into the intraperitoneal cavity is the most predominant route for the formation of metastatic diseases [2]. Hematogenous and lymphatic spread also occurs, yet its clinical significance has not been realized until recently. The evidences of retroperitoneal, submesothelial, and distant metastases cannot be explained by transcoelomic dissemination [3]. The identification of the circulating tumor cells (CTCs) in the blood samples of ovarian cancer patients further supports the role of the hematogenous route in seeding distant metastases [4]. In addition, lymph node metastasis has been already included in the International Federation of Gynecology and Obstetrics (FIGO) ovarian cancer staging system, with its correlation with poor prognosis being well-documented [5].

Lymphovascular space invasion (LVSI), describing the presence of tumor cells within the lumen of lymphatic or vascular capillaries of the primary tumor, is demonstrated to associate with worse clinical outcomes in patients with ovarian cancer [6, 7]. As the first step of dissemination of malignant cells into blood or lymph circulation, LVSI is suggestive of an increased risk of hematogenous and lymphatic metastasis. Since malignant cells may metastasize via multiple routes simultaneously, clinical data regarding hematogenous and lymphatic spread are extensively limited with various confounding factors. Besides, relevant experimental models such as the parabiosis model [8] are cumbersome and hard to accomplish. As a result, the mechanism underlying this novel metastatic route of ovarian cancer, although clinically relevant, still remains elusive. Therefore, we sought to develop gene signatures related to hematogenous and lymphatic metastasis of ovarian cancer, mainly based on the transcriptome profiles with available information on LVSI status. The underlying biological rationale of the identified signature was further explored, which may facilitate the development of predictive biomarkers as well as targeted therapeutic strategies in the near future.

## Methods

### Datasets and data preprocessing

Microarray transcriptome profiles from TCGA, GSE26712, GSE9891, GSE49997 and the corresponding clinical metadata were downloaded from the curatedOvarianData database [9], in which all expression data had been uniformly preprocessed and normalized. The rest of the expression profiles used in the present study, namely GSE2109, GSE30587, GSE40595, GSE40266, were download from Gene Expression Omnibus (http://www.ncbi.nlm.nih.gov/geo) and preprocessed by the robust multi-array average algorithm (RMA). Clinical and pathological characteristics of the cohorts of patients analyzed in this study was listed in Additional file 1: Table S1.

### Differential expression analysis, gene set enrichment analysis (GSEA), and gene set variation analysis (GSVA)

Differentially expressed genes (DEGs) were identified using the limma package. The Benjamini-Hochberg multiple comparison adjustment was applied and the adjusted *P* value < 0.05 was considered statistically significant. Functional annotation was accomplished through the enrichment of Gene Ontology (GO) terms and Kyoto Encyclopedia of Genes and Genomes (KEGG) pathways [10]. GSEA analysis was performed using the Broad Institute desktop application (http://software.broadinstitute.org/gsea/downloads.jsp). Genesets were downloaded from the molecular signatures database (http://software.broadinstitute.org/gsea/msigdb/index.jsp). Sample-wise gene set enrichment scores were generated using the GSVA package [11].

### Tumor purity analysis and correlation analysis

ESTIMATE method [12] was performed to predict tumor purity and the infiltrating level of non-tumor cells. For samples from TCGA dataset, tumor purity inferred by the ABSOLUTE algorithm, another validated approach based on somatic DNA alterations, was obtained from the TCGA working group [13]. The absolute abundance of multiple immune and non-immune stromal populations was inferred by the MCP-counter [14]. The purity-corrected partial Spearman’s correlation between the individual gene expression and immune cell infiltration was generated from the scatter plots obtained in the TIMER database [15]. Spearman’s correction were analyzed in SPSS 25.0.

### Survival analysis

To evaluate the prognostic value of the individual gene expression, we performed a meta-analysis of transcriptome profiles using the curatedOvarianData package. The hazard ratio (HR) with 95% confidence intervals and log-rank *P*-value were obtained from the forest plots. The overall prognostic value of the gene signature was validated in four largest independent datasets with most comprehensive clinical information (TCGA, GSE9891, GSE26712, GSE49997). In each dataset, patients were divided into a high-score and a low-score group based on the best cutoff value generated from the receiver operating characteristic curve (ROC curve). Overall survival curves were calculated using the Kaplan–Meier method, and statistical significance was assessed using the log-rank test. The workflow of this research was summarized in Additional file 1: Table S2.

### Statistical analysis

For normally distributed data, Student t test was used to determine the statistical significance of differences. The Mann-Whitney U test was used for nonparametric data. *P* value < 0.05 was considered statistically significant. All statistical tests were two-sided.

## Results

### Identification of the genes related to lymphovascular metastasis

**Fig. 1:**
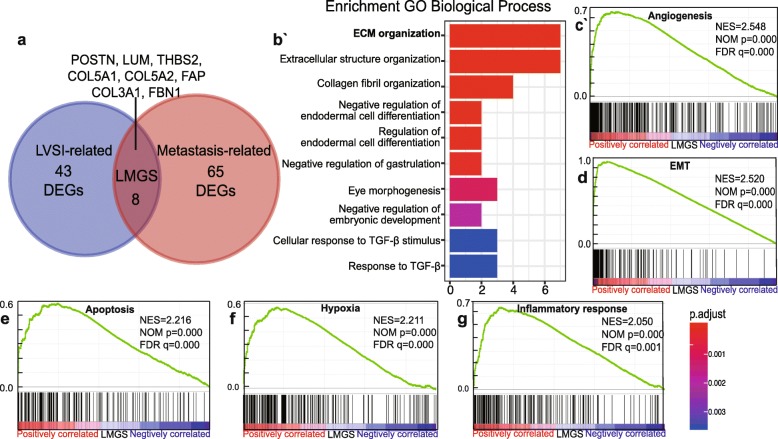
Identification and functional annotation of the gene signature associated with lymphovascular metastasis **a** Venn diagram showed that eight genes were common to the DEGs associated with LVSI status and the DEGs related to metastasis, representing genes potentially correlated with lymphovascular metastasis in ovarian cancer. **b** Functional annotation revealed that the LMGS was closely related to ECM organization. **c-g** Pathways correlated with cancer progression were significantly enriched in the LMGS overexpression group

We firstly defined LVSI status based on the information of lymphatic invasion and venous invasion available in TCGA clinical metadata. Patients with either lymphatic invasion positive or venous invasion positive were regarded as LVSI-positive. Those absent of both types of invasions were defined as LVSI-negative. Differential expression analysis was performed to identify LVSI-associated genes in ovarian cancer, using the transcriptome profiles of 136 LVSI-positive and 56 LVSI-negative samples. DEGs related to metastasis were obtained by analyzing the transcriptome data of high-grade serous ovarian cancer samples from GSE2109. There were eight significantly up-regulated DEGs (POSTN, LUM, THBS2, COL3A1, COL5A1, COL5A2, FAP, FBN1) common in both datasets (Fig. 1a, Additional file 1: Table S3). When validated in another independent dataset GSE30587, all the identified DEGs were significantly elevated in omental metastases compared with the paired primary ovarian tumors (Additional file 2: Figure S1a). Therefore, the eight genes associated with both LVSI status and metastasis were identified as a candidate geneset suggestive of lymphovascular metastasis, hereafter referred to as the Lymphovascular Metastasis Gene Signature (LMGS). Interestingly, according to the expression profiling based on the parabiosis model of ovarian cancer hematogenous metastasis, four genes (POSTN, LUM, COL3A1, COL5A2) of the LMGS were shown to be significantly up-regulated in the omental metastases generated through a hematogenous route (Additional file 2: Figure S1b). This result further indicates the potential role the LMGS in the hematogenous spread of ovarian cancer.

In order to explore the biological rationale of the LMGS, GO term enrichment analysis was performed [10] showing a strong association of the LMGS with extracellular matrix (ECM) organization (Fig. 1b). Protein–protein interaction (PPI) analysis also revealed that the LMGS was closely connected in a biological functional network (Additional file 2: Figure S1c), rather than a randomly combined gene panel.

Considering that the dysregulation of ECM correlates with poor prognosis in multiple cancer types including ovarian cancer [16], we then conducted GSEA analysis to investigate whether the overexpression of LMGS was linked to the key biological traits suggestive of cancer progression. Serous ovarian cancer samples obtained from TCGA dataset were dichotomized based on the expression level of the LMGS (gene Z-score cutoff of + 1) [16]. In the LMGS overexpression group, the majority of the genesets correlated with the hallmarks of cancer were significantly enriched, including angiogenesis, epithelial–mesenchymal transition (EMT), apoptosis, hypoxia, and inflammatory response (Fig. 1c-g).

### The LMGS is predominantly expressed by tumor stromal components

Since all eight genes of the LMGS were closely connected in terms of the biological function, the overall upregulation degree of the LMGS in different samples was compared in addition to the expression levels of an individual gene. Here, we applied the GSVA algorithm to generate a sample-wise enrichment score of the LMGS (referred as LMGS score) as described previously [11]. As an unsupervised and non-parametric gene set enrichment (GSE) analysis of a single-sample expression profiling, the GSVA algorithm outperforms the classic GSE methods such as ssGSEA and PLAGE, providing higher statistic power to detect subtle activation changes [11].

**Fig. 2:**
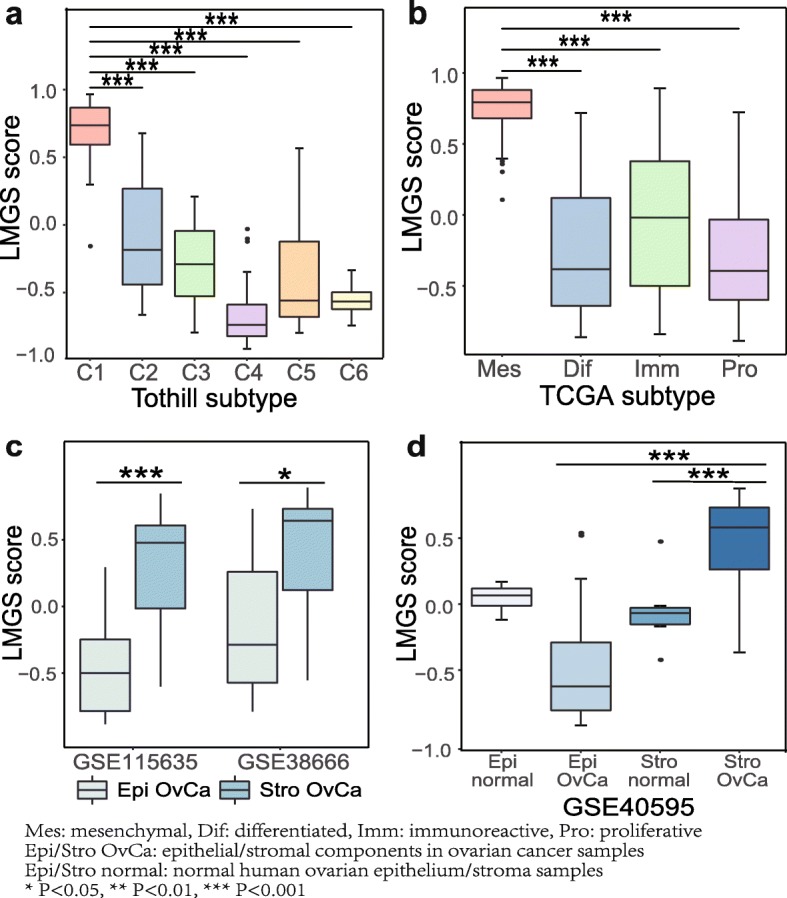
The up-regulation of the LMGS is associated with stromal infiltration. The C1 molecular subtype of **a** the Tothill dataset and **b** TCGA mesenchymal subtype showed significant activation of the LMGS. **c-d** The LMGS was remarkably activated in ovarian cancer stroma in microdissected ovarian cancer samples from three independent datasets

Molecular subtyping often provides clues to the underlying mechanism of a certain phenotype. Hence, we investigated the association between the overexpression of the LMGS and the previously identified molecular subtypes of ovarian cancer. Notably, the LMGS was significantly enriched in the C1 subtype clustered in the Tothill dataset (Fig. 2a and Additional file 3: Figure S2a), a subtype characterized by stromal activation and extensive desmoplasia [17]. Besides, similar analysis demonstrated that the upregulation of the LMGS related with the TCGA mesenchymal subtype (Fig. 2b and Additional file 3: Figure S2b), which displayed high infiltration levels of stromal cells as well as the worst survival [18].

Therefore, we hypothesized that the LMGS overexpression as well as the process of lymphovascular metastasis might at least partly relate to the transcriptional traits dominated by non-epithelial components of tumor tissues. Therefore, the expression pattern of the LMGS was analyzed in three independent transcriptome datasets of microdissected ovarian cancer samples. As expected, the LMGS score was significantly higher in tumor stroma in comparison to epithelial components of ovarian cancer (Fig. 2c, Additional file 3: Figure S2c and S2d), demonstrating that the high level of the LMGS is predominantly attributed to tumor stromal components. The analysis of the dataset GSE40595 additionally suggests that the LMGS may be responsible for the transition of stromal components in carcinogenesis of ovarian cancer, as the LMGS expression was elevated in tumor stroma compared with normal ovarian stroma (Fig. 2d and Additional file 3: Figure S2e).

### Both quantitative and qualitative changes in ovarian cancer stroma are responsible for the increased expression of the LMGS

**Fig. 3:**
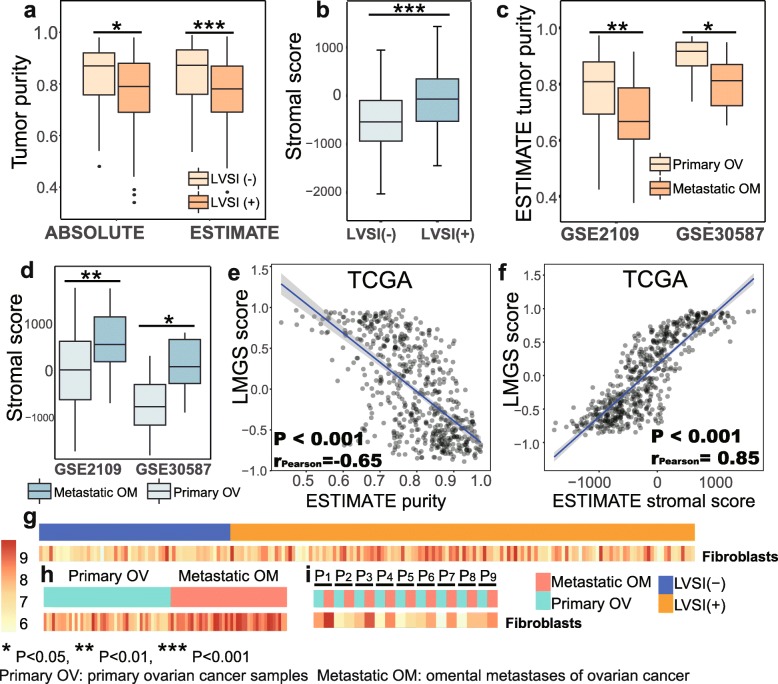
LVSI-positive primary tumors resemble metastatic lesions, to which the infiltration of stromal cells contributes most. LVSI-positive samples were characterized by **a** reduced tumor purity and **b** remarkably elevated mesenchymal infiltration. Omental metastases displayed a similar trend compared to primary ovarian tumors **c-d**. The activation of the LMGS was **e** significantly negatively correlated with tumor purity and **f** positively correlated with mesenchymal infiltration in serous ovarian cancer samples from TCGA dataset. **g** The infiltration of fibroblasts was remarkably elevated in primary ovarian cancer samples with LVSI-positive status. A similar trend was observed in omental metastases compared with primary lesions in **h** dataset GSE2109 and was validated in **i** paired samples from dataset GSE30587

Expression profiles from most public datasets were generated from bulk tumor samples with various degrees of stromal infiltration. To better decipher the role of tumor stroma in the process of lymphovascular metastasis, we applied the ESTIMATE algorithm to analyze tumor purity inferred by the infiltration levels of non-tumor cells. Strikingly, reduced tumor purity as well as elevated mesenchymal infiltration with statistical significance was observed in LVSI-positive samples compared with LVSI-negative ones (Fig. 3a and b). Tumor purity predicted by an alternative approach known as the ABSOLUTE algorithm produced consistent results. Besides, omental metastases displayed similar trends in comparison to primary ovarian tumors (Fig. 3c and d) according to two independent datasets (GSE2109, GSE30587). Taken together, the primary tumors with LVSI-positive status biologically resemble to metastatic tumors in terms of the expression pattern of the LMGS, which is potentially ascribed to the increased proportion of tumor stroma.

Validated in three large independent datasets (TCGA, GSE9891, GSE26712), a remarkable negative correlation was observed between the expression of the LMGS and tumor purity in serous ovarian cancer samples (Fig. 3e, Additional file 4: Figure S3a-S3b). Considering that stromal cells and immune cells are both major cellular components of tumor stroma, we then tested whether the infiltration of immune cells was involved in the expression changes of the LMGS. Although statistic significant, the correlation between the LMGS scores and the ESTIMATE immune scores, which represent the infiltration levels of immune cells, was relatively weak to draw powerful conclusions (Additional file 4: Figure S3c-S3e). The purity-corrected correlation analysis generated from the TIMER database also showed similar results (Additional file 1: Table S4). The correlations between the individual gene expression of the LMGS and the abundance of immune infiltrates, including B cells, CD4+ T cells, CD8+ T cells, neutrophils, macrophages, and dendritic cells, were all significant but extensively weak in ovarian cancer samples. By contrast, the activation of the LMGS in serous ovarian cancer samples from three datasets was strongly and positively correlated with the presence of stromal cells represented by ESTIMATE stromal scores (Fig. 3f, Additional file 4: Figure S3f-S3 g). Moreover, quantification of eight immune and two stromal cell populations inferred by the MCP-counter revealed that infiltration of fibroblasts was remarkably elevated in primary ovarian cancer samples with LVSI-positive status compared to the LVSI-negative ones (Fig. 3g, Additional file 4: Figure S3 h). Similar results were obtained when exploring the infiltration patterns in omental metastases compared with primary ovarian tumors (Fig. 3h-i, Additional file 4: Figure S3i-S3j). These data indicate that the increased infiltration of stromal cells, especially fibroblasts, is responsible for the reduction of tumor purity in the process of lymphovascular metastasis.

**Fig. 4:**
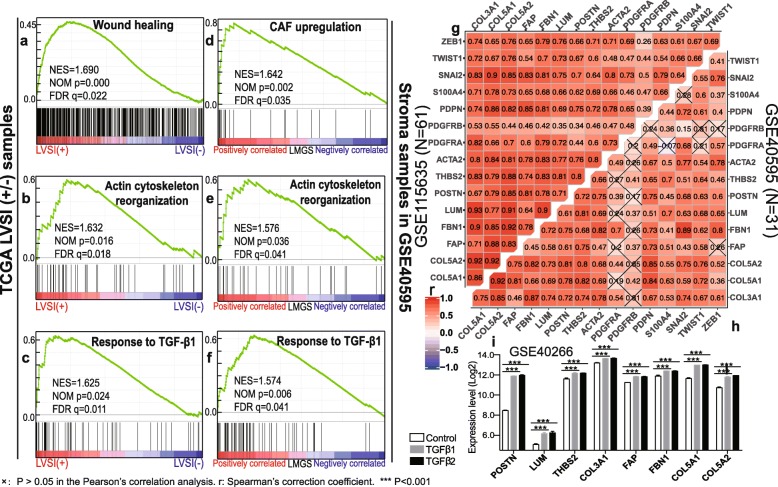
The activation of CAFs contributes to the overexpression of the LMGS in lymphovascular metastasis. **a-c** GSEA plots showed that pathways representative of CAF activation were significantly enriched in LVSI-positive samples, as well as in stromal profiles with high levels of the LMGS in the dataset GSE40595 **d-f**. The Pearson’s correlation analysis between the expression of the identified genes and CAF-specific markers was conducted based on the transcriptome profiles of ovarian cancer stroma in dataset **g** GSE115635 and **h** GSE40595. **i** All the identified genes were significantly unregulated in the ovarian fibroblasts NOF151-hTERT treated with either TGF-β1 or TGF-β2 compared to controls, according to the expression profiles in the dataset GSE40266

As tumor stromal activation involves both quantitative and qualitative changes of stromal components, we then investigated whether the overexpression of the LMGS also reflected the activation of tumor stroma related to cancer-associated fibroblasts (CAFs). GSEA was conducted revealing that in LVSI-positive samples, gene signatures representative of wound healing were enriched (Fig. 4a-c), a process where myofibroblasts resembling CAFs are recruited and reprogrammed [19]. In the stromal profiles of the dataset GSE40595, pathways involved in CAF activation were also shown to be positively correlated to the upregulation of the LMGS (Fig. 4d-f). Besides, based on in the transcriptome profiles of the ovarian cancer stroma from two independent datasets, a significant and positive correlation was validated between the expression of the eight genes in the LMGS and several common CAF markers (Fig. 4g and Additional file 5: Figure S4a).

Of note, GO annotation as well as GSEA uncovered a potential link between the upregulation of the LMGS and the activation of the TGF-β signaling pathway (Fig. 1b and c), the role of which in CAF activation has been validated in several cancer types including ovarian cancer [20, 21]. Hence, we investigated whether the expression of the LMGS was partly regulated by the TGF-β pathway. Strikingly, according to the transcriptome profiles in the dataset GSE40266, all eight genes of the LMGS were significantly unregulated in the ovarian fibroblasts NOF151-hTERT treated with either TGF-β1 or TGF-β2 compared to controls (Fig. 4i). Together, these data suggest that in addition to the increased infiltration of the ovarian cancer stroma, the activation of CAFs also contributes to the overexpression of the LMGS in lymphovascular metastasis.

To further investigate the effects of the LMGS overexpression in ovarian cancer cells, we ranked the ovarian cancer cell lines in the Cancer Cell Line Encyclopedia (CCLE) database by the GSVA scores of the LMGS. Top-ranked cell lines displayed a mesenchymal phenotype based on the EMT phenotype annotation (Additional file 1: Table S5) generated from published sources [22], implicating that ovarian cancer cells with high levels of the LMGS expression may acquire invasive phenotypes partly through EMT.

### The overexpression of the LMGS is associated with increased invasiveness and poor prognosis in patients with serous ovarian cancer

The activation of tumor stroma is considered as an important determinant of poor survival in patients with solid tumors including ovarian cancer, we next evaluated the prognostic values of the LMGS in patients with serous ovarian cancer.

**Fig. 5:**
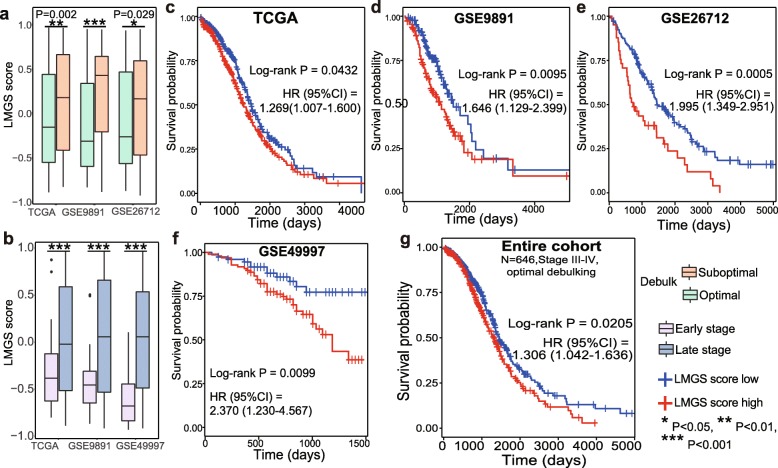
The LMGS up-regulation is associated with poor prognosis in patients with serous ovarian cancer. The LMGS was significantly enriched in **a** patients undergoing suboptimal cytoreduction and **b** those with late-stage disease. The prognostic significance of the LMGS in patients with serous ovarian cancer was validated in dataset **c** TCGA (*N* = 557), **d** GSE9891 (*N* = 240), **e** GSE26712 (*N* = 185), **f** GSE49997 (*N* = 171). **g** Survival analysis across the four validation datasets (*N* = 646) was conducted in patients with late-stage disease undergoing optimal cytoreduction

In three large validation datasets, the LMGS was significantly upregulated in patients undergoing suboptimal cytoreduction (Fig. 5a, Additional file 5: Figure S4a-S4c). Late-stage patients also displayed higher expression levels of the LMGS compared to those with stage I-II diseases (Fig. 5b, Additional file 5: Figure S4d-S4f). These data uncovered the correlation between the LMGS overexpression and the increased invasiveness of serous ovarian cancer, which notoriously relates to poor survival.

In order to evaluate the prognostic values of the individual gene expression of the LMGS, we conducted a meta-analysis of the transcriptome profiles from multiple public datasets. According to the hazard ratio (HR) generated from the forest plots, high expression of any gene of the LMGS was significantly correlated with worse overall survival (OS). These results remained significant when adjusted for tumor stage and debulking status. Moreover, similar results were obtained in the analysis of progression-free survival (PFS) (Table 1), suggesting that the prognostic effects of the eight genes in both OS and PFS are independent from these clinical parameters.

**Table 1:** Up-regulation of the identified genes independently associate with poor survival in ovarian cancer patients

Gene	OS		Adjusted OS		PFS		Adjusted PFS	
	HR (95%CI)	*P*	HR (95%CI)	*P*	HR (95%CI)	*P*	HR (95%CI)	*P*
POSTN	1.16 (1.09, 1.22)	4.56E-07	1.09 (1.02, 1.16)	0.0137	1.14 (1.06, 1.22)	0.0003	1.09 (1.01, 1.17)	0.0349
LUM	1.18 (1.12, 1.25)	2.67E-09	1.10 (1.03, 1.18)	0.0039	1.17 (1.10, 1.26)	6.17E-06	1.12 (1.04, 1.21)	0.0043
THBS2	1.22 (1.15, 1.30)	3.59E-10	1.13 (1.05, 1.21)	0.0008	1.21 (1.12, 1.29)	2.77E-07	1.13 (1.05, 1.23)	0.0023
COL3A1	1.18 (1.12, 1.25)	1.46E-08	1.10 (1.03, 1.18)	0.0053	1.20 (1.12, 1.29)	2.05E-07	1.14 (1.05, 1.23)	0.0010
COL5A1	1.20 (1.14, 1.27)	6.31E-11	1.13 (1.05, 1.21)	0.0005	1.17 (1.09, 1.25)	8.38E-06	1.11 (1.03, 1.20)	0.0091
COL5A2	1.20 (1.14, 1.27)	3.15E-11	1.12 (1.04, 1.19)	0.0015	1.18 (1.10, 1.27)	1.70E-06	1.12 (1.03, 1.21)	0.0047
FAP	1.20 (1.14, 1.27)	2.52E-11	1.13 (1.06, 1.21)	0.0003	1.18 (1.10, 1.26)	1.61E-06	1.13 (1.05, 1.22)	0.0019
FBN1	1.19 (1.12, 1.25)	2.93E-09	1.12 (1.05, 1.19)	0.0010	1.13 (1.05, 1.23)	1.71E-06	1.18 (1.10, 1.27)	0.0013

Adjusted OS/adjusted PFS: results were adjusted for tumor stage and debulking status

Next, four largest cohorts with most comprehensive clinical information from independent datasets were used to validate the overall prognostic value of the LMGS in patients with serous ovarian cancer. It was confirmed that patients with high levels of the LMGS had worse overall survival in each validation dataset (Fig. 5c-f). When applied to the entire cohort combining the above validation datasets, the overexpression of the LMGS was further shown to be an independent predictor of OS (log-rank *P* = 0.0205, HR = 1.306[1.042–1.636]) in patients with late-stage diseases undergoing optimal cytoreduction (Fig. 5g).

## Discussion

Unlike transcoelomic metastasis of ovarian cancer, limited experimental models [23] and clinical data regarding hematogenous and lymphatic spread results in a less comprehensive understanding of its underlying mechanisms, as well as a possible underestimation of its clinical significance. Although distant metastases generated by hematogenous and lymphatic routes may hardly cause immediate death, their presence is significantly correlated with poorer prognosis [2, 24]. With the continuous progress in the treatment of ovarian cancer, prolonged survival allows more time for the development of distant metastases, which becomes clinically significant particularly for long-term survivors [24]. In addition, ovarian cancer cells are demonstrated to metastasize hematogenously with a strong predilection for the omentum [8], the most common site of ovarian cancer metastasis, suggesting that hematogenous spread may also plays an important role in the formation of intraperitoneal metastases. Experimentally, the ErbB3/NRG1 signaling axis is identified as a dominant pathway responsible for hematogenous omental metastasis using a well-designed parabiosis model [8]. Downregulation of CXCR4 also resulted in a robust reduction of the circulating ovarian cancer cells, suggesting a possible role of the SDF1/CXCR4 axis in the hematogenous route of dissemination [25]. In terms of lymphatic metastasis, molecules including USP7, FAK, as well as the VEGFC-VEGFR3 interaction, are shown to associate with incidence of lymph node metastases of ovarian cancer [2].

The gene signature identified in the present study provides a novel insight into the potential biological processes and cell types involved in hematogenous and lymphatic metastasis of ovarian cancer. We highlight that both quantitative and qualitative changes in ovarian cancer stroma are involved in stromal activation. Notably, by comparing the transcriptional levels of the stromal marker vimentin and the reactive stroma marker ACTA2, Zhenqiu Liu et al. [26] proposed that changes of the epithelium-to-stroma ratio were modest and less significant than qualitative changes in the process of stromal activation. Similar results were reported by Dong-Joo Cheon et al. [27] using an additional epithelial marker EPCAM. Alternatively, tumor purity analysis in our work indicates that increased infiltration of stromal cells is also a distinct feature in reactive stroma. The abundance of non-tumor cells inferred by the ESTIMATE and the MCP-counter, each algorithm based on a panel of over 100 transcriptomic markers with confirmed robustness and sensitivity [12, 14], reveals that the elevated infiltration of fibroblasts contributes to the dynamic changes of tumor purity in stromal activation. Since CAFs are heterogeneous population lacking a universal marker with high sensitivity and specificity [28], a group of CAF-specific markers was chosen [29] to identify the correlation between CAFs activation and the overexpression of the identified gene signature. In parallel with the results of GSEA, the overexpression of the LMGS is positively correlated with the activation of CAFs in ovarian cancer stroma. Therefore, it is rational to speculate that both the recruitment and the activation of CAFs in tumor stroma are major processes involved in lymphovascular metastasis of ovarian cancer.

It is widely-accepted that stromal cells, especially CAFs, dominate in the tumor microenvironment (TME) and possess multiple tumor-promoting functions, facilitating tumor invasion and metastasis through direct and indirect crosstalk with tumor cells as well as with other non-tumor components like immune cells and endothelial cells [30].

Firstly, the co-evolution of cancer cells and CAFs facilitate tumor growth and spread via multiple interactions. CAFs produce autocrine and paracrine cytokines, chemokines and growth factors like CXCL1, CCL5, HB-EGF and TGF-α, subsequently promoting cancer cell invasiveness through upregulation of matrix metalloproteinases (MMPs) and induction of EMT. CAFs can additionally reprogram ovarian cancer cell metabolism via producing metabolites or altering the key enzymatic activities [31]. In turn, ovarian cancer cells as well as non-tumor components like tumor-associated macrophages (TAMs), secrete various factors including TGF-β, IGFs and PDGF to accelerate NF-CAF transition and maintain CAF activation [29], which keep providing a tumor-promoting milieu.

Besides, angiogenesis and inflammatory response are involved in the tumor-stroma crosstalk, which was also revealed in the above enrichment analysis. Pathological angiogenesis, supplying adequate nutrients and oxygen to tumor cells, often indicates the metastatic potential in tumors. CAFs secretes key pro-angiogenic factor VEGF-A as a result of HOXA9 upregulation from ovarian cancer cells, promoting proliferation and invasiveness of endothelial cells so as to trigger angiogenesis [32]. Many of the pro-inflammatory factors including CXCL12, IL-6, and COX-2 produced by CAFs are also shown to recruit and mobilize endothelial cells for de novo angiogenesis [33, 34]. Of note, Sonic Hedgehog (SHH) secreted from ovarian cancer cells activates Hh signaling in CAFs, inducing VEGF-C expression and promoting lymphangiogenesis in vitro and in vivo [35, 36]. These results elucidate potential mechanisms by which CAFs constitute a supportive niche for lymphovascular metastasis and cancer progression in ovarian cancer.

Referred to as “wounds that never heal”, cancer and chronic inflammation are closely correlated. CAFs overexpress pro-inflammatory chemokines and cytokines such as IL-6, COX-2, and CXCL1, which mediate tumor-related inflammation and induce carcinogenesis [34]. These CAF-derived immunomodulatory factors also promote the recruitment of regulatory T cells and TAMs, both of which are essential in support of immune escape during tumor metastasis. In addition, CAFs promote the invasiveness and adhesion of monocytes by up-regulating IL-8, which subsequently induces M2 polarization of macrophages. TAMs further synergize with CAFs to suppress the function of natural killer (NK) cells, maintaining a pre-metastatic niche with impaired immune surveillance [37].

Considering the essential role of tumor stroma in promoting cancer progression, strategies targeting stromal cells exert great potential in improving clinical outcomes of ovarian cancer patients. Notably, low resistance rate is a distinct advantage of anti-stromal therapies, largely relying on the genetic stability of stromal cells. Among the genes identified in the present study, the safety of FAP-antibody sibrotuzumab has been validated in phase I trials in patients with tumors highly expressing FAP [38]. Although beneficial effects were not observed in a phase II trial for metastatic colorectal cancer [39], the efficacy of RO6874281 (an FAP-antibody fused with IL-2) is currently under clinical evaluation (NCT03424005, NCT03193190, NCT03386721, NCT02627274, NCT03063762, NCT03875079).

In addition, TGF-β-targeted therapies are currently investigated as a promising approach to impede cancer progression, since TGF-β has been demonstrated as the key driver of fibroblast activation and CAF formation in different cancer types. In line with the available researches [27, 40], some of the validated TGF-β downstream effectors including FAP, POSTN, VCAN, COL5A1 and THBS2 were also shown to be relevant to hematogenous and lymphatic spread in our study. In ovarian cancer, the activation of TGF-β-dependent and TGF-β-independent Smad pathways reprograms fibroblasts by inducing various matrix proteins to acquire CAF properties [41]. These CAF-related molecules further confer the aggressiveness of ovarian cancer cells through the activation of NF-κB signaling pathway and the up-regulation of CD44, MMP9 and hyaluronan-mediated motility receptor (HMMR) [40].

Currently, there are over 50 clinical trials evaluating TGF-β-targeted therapies in cancers [42]. For patients with advanced-stage ovarian cancer, a bi-shRNAfurin/GMCSF-expressing autologous tumor cell (FANG) vaccine capable of decreasing TGFβ1/2 protein expression, was demonstrated to improve immune response and recurrence free survival in a phase II trial. The efficacy of FANG vaccine as maintenance (NCT01309230, NCT02346747) or adjuvant therapy (NCT02725489, NCT03073525, NCT01551745, NCT01867086) is currently under investigation in phase II trials. Moreover, normalization of TME is likely to be a preferable approach for stroma-targeted therapy, since obliterating tumor stroma altogether often enhances the aggressiveness of cancer cells [30].

Of note, some genes we explored overlap with previously identified gene signatures associated with cancer progression and poor survival in various solid tumors including ovarian [27, 43], breast [44], and colorectal cancer [45], suggesting that the abnormal expression of these genes might be a potential indicator of aggressive behavior across cancer types. One major strength of the present study is that our findings are cross-validated in multiple independent datasets, indicating that the results may at least partly reflect some intrinsic features of ovarian cancer metastasis, rather than a random event or a technical artifact. Besides, the prognostic significance and underlying mechanisms of the lymphovascular metastasis remained elusive largely due to the limited clinical data and cumbersome experimental models. The present study provided a thorough understanding of the potential mechanisms though which ovarian cancer cells confer the lymphovascular metastatic phenotype via the up-regulation of a gene signature dominated in tumor stroma. We elaborated the essential role of CAFs in the process of hematogenous and lymphatic metastasis of serous ovarian cancer innovatively through bioinformatic analyses. It is, to our knowledge, the first bioinformatic research in this area, which supplemented the previous experimental and clinical researches. As these results are generated by bioinformatic methods, we acknowledge that the functions and the underlying mechanisms of the identified gene signature need further exploration before translation to the clinic. With further validation and optimization, we anticipate that these identified genes will not only work as potential biomarkers to predict metastasis and prognosis in ovarian cancer patients, but also facilitate the development of targeted therapies to improve clinical outcomes of this lethal disease.

## Conclusions

In this manuscript, we identified a panel of genes closely correlated with hematogenous and lymphatic metastasis of serous ovarian cancer. The upregulation of this gene signature, predominantly expressed by the increased infiltration of reactive stroma, is further confirmed to associate with tumor invasiveness and poor survival. Utilizing multiple transcriptome profiles available in the public database, we explored the potential biological rationale underlying this relatively novel metastatic route of ovarian cancer, highlighting the imperative role of tumor stroma in the development of ovarian cancer metastasis.

## Supplementary information

**Additional file 1: Table S1.** Clinical and pathological characteristics of the cohorts of patients analyzed in the manuscript. **Table S2.** Flowchart of this study. **Table S3.** Eight genes common to both LVSI- and metastasis-related DEGs were listed. Fold changes and adjusted *P* values were generated by limma package. **Table S4.** Results of purity-corrected correction analysis from TIMER database showed a significant but weak correlation between the expression levels of the identified genes and the infiltration of immune cells in ovarian cancer samples. **Table S5.** The expression of the LMGS in the ovarian cancer CCLE cell lines were ranked, with the corresponding EMT phenotypes annotated based on two public sources

**Additional file 2: Figure. S1.** (a) Paired t-test revealed that all eight genes were significantly elevated in omental metastases compared with the corresponding primary ovarian tumors in the dataset GSE30587. (b) Four genes (POSTN, LUM, COL3A1, COL5A2) of the LMGS were remarkably elevated, while COL5A1 was significantly down-regulated in SKOV3-OM3 (subpopulations derived from omental tumors in guest mice of the parabiosis models, representing omental metastases generated through a hematogenous route), compared to SKOV3ip1 intraperitoneal injected to the host mice (representing the primary tumors) in the dataset GSE52999. (c) Genes of the LMGS were likely to form a biologically functional network based on PPI analysis. Primary OV: primary ovarian cancer samples, Metastatic OM: omental metastases of ovarian cancer. * *P* < 0.05, ** *P* < 0.01, *** *P* < 0.001

**Additional file 3: Figure. S2.** All eight genes of the LMGS were highly enriched in the C1 molecular subtype of (a) the Tothill dataset and (b) in the TCGA mesenchymal subtype. (c) The expression levels of individual gene of the LMGS were significantly elevated in tumor stroma compared with the epithelial components in dataset GSE38666. A similar tendency was observed in paired samples in (d) dataset GSE115635, as well as in stromal components compared with normal ovarian stroma in (e) dataset GSE40595.

**Additional file 4: Figure. S3.** The significant and negative correlation between the activation of the LMGS and tumor purity was validated in (a) GSE9891 and (b) GSE26712. (c-e) The positive correlation between the expression of the LMGS and immune cell infiltration was significant but relatively weak. The activation of the LMGS was positively correlated with mesenchymal infiltration in serous ovarian cancer samples from (f) GSE9891 and (g) GSE26712. The infiltration of immunocytes was similar between the primary ovarian cancer samples with LVSI-positive status versus LVSI-negative ones. A similar trend was observed in omental metastases compared with primary lesions in (**h**) dataset GSE2109 and was validated in (**i**) paired samples from dataset GSE30587

**Additional file 5: Figure. S4.** Genes of the LMGS were remarkably elevated in **(a-c)** patients undergoing suboptimal cytoreduction and (**d-f)** those with late-stage serous ovarian cancer

## Data Availability

The datasets analyzed during the current study are available in the curatedOvarianData database [9], and Gene Expression Omnibus (http://www.ncbi.nlm.nih.gov/geo).

## Abbreviations

ACTA2: Actin alpha 2, smooth muscle
CAFs: Cancer-associated fibroblasts
CCL5: C-C motif chemokine ligand 5
CCLE: The Cancer Cell Line Encyclopedia
COL3A1: Collagen type III alpha 1 chain
COL5A1: Collagen type V alpha 1 chain
COL5A2: Collagen type V alpha 2 chain
CTCs: Circulating tumor cells
CXCL1: C-X-C motif chemokine ligand 1
DEGs: Differentially expressed genes
ECM: Extracellular matrix
EMT: Epithelial–mesenchymal transition
EPCAM: Epithelial cell adhesion molecule
FAP: Fibroblast activation protein alpha
FBN1: Fibrillin 1
FIGO: International Federation of Gynecology and Obstetrics
GO: Gene ontology
GSEA: Gene set enrichment analysis
GSVA: Gene set variation analysis
HB-EGF: Heparin-binding epidermal growth factor
HMMR: Hyaluronan-mediated motility receptor
HR: Hazard ratio
IGF: Insulin growth factor
KEGG: Kyoto Encyclopedia of Genes and Genomes
LUM: Lumican
LVSI: Lymphovascular space invasion
MMPs: Matrix metalloproteinases
NF: Normal fibroblast
OS: Overall survival
PDGF: Platelet-derived growth factor
PFS: Progression free survival
POSTN: Periostin
PPI: Protein–protein interaction
RMA: Robust multi-array average algorithm
ROC curve: Receiver operating characteristic curve
TAMs: Tumor-associated macrophages
TCGA: The Cancer Genome Atlas
TGF-β: Transforming growth factor-β
THBS2: Thrombospondin 2
TME: Tumor microenvironment
TNF-α: Tumor necrosis factor-α
