# Supplementary material for: Gene signature characteristic of elevated stromal infiltration and activation is associated with increased risk of hematogenous and lymphatic metastasis in serous ovarian cancer
Source: BMC Cancer. 2019 Dec 30;19:1266. doi: 10.1186/s12885-019-6470-y (PMC6937680; doi:10.1186/s12885-019-6470-y)
Supplement: Supplementary file 1 — Additional file 1: Table S1. Clinical and pathological characteristics of the cohorts of patients analyzed in the manuscript. Table S2. Flowchart of this study. Table S3. Eight genes common to both LVSI- and metastasis-related DEGs were listed. Fold changes and adjusted P values were generated by limma package. Table S4. Results of purity-corrected correction analysis from TIMER database showed a significant but weak correlation between the expression levels of the identified genes and the infiltration of immune cells in ovarian cancer samples. Table S5. The expression of the LMGS in the ovarian cancer CCLE cell lines were ranked, with the corresponding EMT phenotypes annotated based on two public sources [file 12885_2019_6470_MOESM1_ESM.docx]

**Table S1** Clinical and pathological characteristics of the cohorts of patients analyzed in the manuscript.

| **GSE9891** | | **TCGA** | | **GSE49997** | | **GSE26712** | | **GSE140082** | |
| --- | --- | --- | --- | --- | --- | --- | --- | --- | --- |
|  |  |  |  |  |  |  |  | **Bevacizumab arm** | |
| **Age** |  | **Age** |  | **Age** |  | **Age** |  | **Age** |  |
| Mean | 60(10.07) | Mean | 59(11.52) | Mean | 58(11.99) | Mean | 61(11.86) | Mean | 58(11.08) |
| Range | 23-80 | Range | 26-89 | Range | 26-85 | Range | 26-84 | Range | 26-80 |
| **Debulking** |  | **Debulking** | | **Debulking** | | **Debulking** | | **Debulking** |  |
| Optimal |  | Optimal | 365 | Optimal | 121 | Optimal | 90 | Optimal | 153 |
| Suboptimal |  | Suboptimal | 138 | Suboptimal | 50 | Suboptimal | 95 | Suboptimal | 45 |
| NA |  | NA | 54 |  |  |  |  | Inoperable | 1 |
| **FIGO stage** | | **FIGO stage** | | **FIGO stage** | | **FIGO stage** | | **FIGO stage** | |
| Early | 21 | Early | 42 | Early | 5 | III | 146 | Early | 27 |
| Late | 218 | Late | 512 | Late | 166 | IV | 36 | Late | 172 |
| NA | 1 | NA | 3 |  |  | NA | 3 | **Histology** |  |
| **Histology** |  | **Histology** |  | **Histology** |  | **Histology** |  | Serous | 150 |
| Serous | 240 | Serous | 557 | Serous | 171 | Serous | 185 | Other | 49 |
| **Grade** |  | **Grade** |  | **Grade** |  | **Grade** |  | **Grade** |  |
| High-grade | 144 | High-grade | 470 | High-grade | 125 | High-grade | 185 | High-grade | 142 |
| Low-grade | 94 | Low-grade | 75 | Low-grade | 45 |  |  | Low-grade | 46 |
| NA | 2 | NA | 12 | NA | 1 |  |  | NA | 11 |
| **Total** | 240 | **Total** | 557 | **Total** | 171 | **Total** | 185 | **Total** | 199 |

**Table S2** Flowchart of this study.

Identification of the LMGS

LVSI-related DEGs

Metastasis-related DEGs

Functional annotation

Expressional pattern

Relation with CAF infiltration

Survival analysis

| **Table S3** Eight genes common to both LVSI- and metastasis-related DEGs, collectively referred to as the LMGS. | | | | | | |
| --- | --- | --- | --- | --- | --- | --- |
| **Gene name** | **Gene symbol** | **LVSI-related** | | | **Metastasis-related** | |
|  |  | **Fold Change** | **adjusted P** | **Fold Change** | | **adjusted P** |
| **Periostin** | **POSTN** | 3.08639 | 0.02320 | 5.11785 | | 0.00048 |
| **Lumican** | **LUM** | 2.50187 | 0.00840 | 2.97323 | | 0.00306 |
| **Thrombospondin 2** | **THBS2** | 2.28793 | 0.01413 | 2.48479 | | 0.01157 |
| **Collagen type V alpha 2 chain** | **COL5A2** | 2.00921 | 0.02320 | 2.05279 | | 0.03919 |
| **Collagen type V alpha 1 chain** | **COL5A1** | 1.77411 | 0.03883 | 2.13162 | | 0.01198 |
| **Fibroblast activation protein alpha** | **FAP** | 1.74877 | 0.03233 | 2.19489 | | 0.01165 |
| **Collagen type III alpha 1 chain** | **COL3A1** | 1.62715 | 0.04999 | 2.10587 | | 0.00210 |
| **Fibrillin 1** | **FBN1** | 1.58607 | 0.01784 | 2.05260 | | 0.01712 |

Note: Fold changes and adjusted P values were generated by limma package.

**Table S4** A significant but weak correlation between the LMGS and the infiltration of immune cells in ovarian cancer samples.

| **Gene** | **Immune cell infiltration** | **r-partial** | **P** |
| --- | --- | --- | --- |
| **POSTN** | B Cell | -0.104775235 | 0.021684597 |
|  | CD8+ T Cell | 0.061753684 | 0.176786013 |
|  | CD4+ T Cell | 0.094409253 | 0.038674804 |
|  | Macrophage | 0.081823986 | 0.073292675 |
|  | Neutrophil | 0.16314724 | 0.00033178 |
|  | Dendritic Cell | 0.162866584 | 0.000339834 |
| **LUM** | B Cell | -0.097241145 | 0.033176404 |
|  | CD8+ T Cell | 0.087496217 | 0.055413075 |
|  | CD4+ T Cell | 0.057660981 | 0.207292849 |
|  | Macrophage | 0.198047111 | 1.24E-05 |
|  | Neutrophil | 0.17055319 | 0.000173734 |
|  | Dendritic Cell | 0.126476859 | 0.005521942 |
| **THBS2** | B Cell | -0.080199806 | 0.079201704 |
|  | CD8+ T Cell | 0.036776831 | 0.421448269 |
|  | CD4+ T Cell | 0.11907871 | 0.009017787 |
|  | Macrophage | 0.094870379 | 0.037730092 |
|  | Neutrophil | 0.131036655 | 0.004030134 |
|  | Dendritic Cell | 0.131170734 | 0.003992399 |
| **COL3A1** | B Cell | -0.146129609 | 0.001325311 |
|  | CD8+ T Cell | -0.051587815 | 0.259304387 |
|  | CD4+ T Cell | 0.036183731 | 0.428980205 |
|  | Macrophage | 0.107378309 | 0.018611896 |
|  | Neutrophil | 0.007860716 | 0.863615762 |
|  | Dendritic Cell | 0.001656902 | 0.971117877 |
| **COL5A1** | B Cell | -0.209494559 | 3.67E-06 |
|  | CD8+ T Cell | -0.123170612 | 0.006896694 |
|  | CD4+ T Cell | 0.013006197 | 0.776241356 |
|  | Macrophage | -0.007518303 | 0.869502373 |
|  | Neutrophil | -0.125963417 | 0.005717808 |
|  | Dendritic Cell | -0.124025296 | 0.006514678 |
| **COL5A2** | B Cell | -0.139434451 | 0.002199656 |
|  | CD8+ T Cell | -0.004045666 | 0.929554501 |
|  | CD4+ T Cell | 0.08977009 | 0.049344108 |
|  | Macrophage | 0.124617374 | 0.006261277 |
|  | Neutrophil | 0.083127368 | 0.068815173 |
|  | Dendritic Cell | 0.078786233 | 0.084653608 |
| **FAP** | B Cell | -0.0878877 | 0.05432609 |
|  | CD8+ T Cell | 0.08605199 | 0.05958055 |
|  | CD4+ T Cell | 0.12909347 | 0.00461445 |
|  | Macrophage | 0.15431356 | 0.00069292 |
|  | Neutrophil | 0.22114981 | 9.92E-07 |
|  | Dendritic Cell | 0.19498021 | 1.69E-05 |
| **FBN1** | B Cell | -0.1096984 | 0.0162006 |
|  | CD8+ T Cell | -0.0186157 | 0.6841384 |
|  | CD4+ T Cell | 0.00131216 | 0.97712528 |
|  | Macrophage | 0.15643534 | 0.0005826 |
|  | Neutrophil | 0.01886454 | 0.68014849 |
|  | Dendritic Cell | 0.02441619 | 0.59360674 |

Note: The above data were generated from the purity-corrected correction analysis in TIMER database.

| **Table S5** The ranking list of the expression of the LMGS in the ovarian cancer CCLE cell lines. | | | | | |
| --- | --- | --- | --- | --- | --- |
| **Cell line** | **LMGS score** | Atlas of Genetics and Cytogenetics in Oncology and Haematology* | Huang et al., 2013 Cell Death and Disease^+^ | | |
|  |  |  |  |  |  |
|  |  | **EMT Phenotype** | **EMT Phenotype** | **Morphology** | **Histology** |
| COV504 | 0.7992 | Mesenchymal |  |  |  |
| SNU840 | 0.7813 |  |  |  |  |
| JHOM1 | 0.7284 |  |  |  |  |
| TOV112D | 0.7042 | Mesenchymal | Mesenchymal | Spindle | Endometrioid |
| COV434 | 0.6511 | Mesenchymal |  |  |  |
| OV7 | 0.6487 | Mesenchymal | Intermediate Mesenchymal | Spindle | Poorly differentiated |
| A2780 | 0.5849 | Mesenchymal | Mesenchymal | Rounded | Undifferentiated |
| JHOS2 | 0.5784 |  | Intermediate Epithelial | Cobblestone | Serous cystadenocarcinoma |
| IGROV1 | 0.5656 | Epithelial | Intermediate Epithelial | Cobblestone/Rounded | Endometrioid |
| EFO27 | 0.5597 | Mesenchymal |  |  |  |
| SKOV3 | 0.4727 |  | Intermediate Mesenchymal | Spindle | Serous |
| COV362 | 0.4071 | Mesenchymal |  |  |  |
| COV644 | 0.3591 | Epithelial |  |  |  |
| ES2 | 0.2682 |  |  |  |  |
| OVMA | 0.2377 | Epithelial |  |  |  |
| OV56 | 0.1679 | Mesenchymal | Intermediate Epithelial | Rounded/Spindle | Poorly differentiated |
| HeyA8 | 0.0787 |  | Mesenchymal | Spindle | Serous |
| OVCAR8 | 0.0598 | Epithelial | Epithelial | Cobblestone | Undifferentiated |
| COV318 | -0.0028 | Epithelial |  |  |  |
| Caov3 | -0.0234 | Epithelial | Epithelial | Cobblestone | Serous |
| NIHOVCAR3 | -0.0356 | Epithelial | Epithelial | Cobblestone | Serous |
| FUOV1 | -0.0391 |  | Intermediate Epithelial | Cobblestone | Serous papillary |
| OVSAHO | -0.0538 | Epithelial |  |  |  |
| OV90 | -0.0583 |  | Epithelial | Cobblestone | Serous |
| EFO21 | -0.0644 |  | Intermediate Epithelial | Cobblestone | Dedifferentiated serous |
| OVKATE | -0.0972 | Epithelial |  |  |  |
| OVK18 | -0.0972 |  | Mesenchymal | Cobblestone/Rounded | Endometrioid |
| OAW28 | -0.1367 | Epithelial | Intermediate Epithelial | Cobblestone | Serous cystadenocarcinoma |
| Caov4 | -0.1481 |  |  |  |  |
| OVCAR4 | -0.1671 | Epithelial |  |  |  |
| COLO704 | -0.2353 | Mesenchymal |  |  |  |
| OVTOKO | -0.2530 | Mesenchymal |  |  |  |
| RMUGS | -0.2560 | Epithelial |  |  |  |
| MCAS | -0.2872 | Epithelial |  |  |  |
| JHOM2B | -0.2968 |  |  |  |  |
| TYKnu | -0.3645 |  | Mesenchymal | Spindle | Undifferentiated |
| RMGI | -0.3692 | Epithelial |  |  |  |
| TOV21G | -0.4010 |  |  |  |  |
| OC 316 | -0.4205 |  |  |  |  |
| SNU119 | -0.4437 |  |  |  |  |
| JHOS4 | -0.4546 |  | Intermediate Epithelial | Cobblestone | Serous |
| JHOC5 | -0.5244 |  |  |  |  |
| OAW42 | -0.5395 | Epithelial | Intermediate Epithelial | Cobblestone | Serous papillary |
| OC 314 | -0.6047 |  |  |  |  |
| Note: the corresponding EMT phenotypes were annotated based on the following public sources:  *: http://atlasgeneticsoncology.org/cell_lines.html  +: Huang RY, Wong MK, Tan TZ, Kuay KT, Ng AH, Chung VY et al: An EMT spectrum defines an anoikis-resistant and spheroidogenic intermediate mesenchymal state that is sensitive to e-cadherin restoration by a src-kinase inhibitor, saracatinib (AZD0530). Cell Death Dis. 2013; 4:e915. | | | | | |
