# Supplementary figures and images for: Gene signature characteristic of elevated stromal infiltration and activation is associated with increased risk of hematogenous and lymphatic metastasis in serous ovarian cancer
Source: BMC Cancer. 2019 Dec 30;19:1266. doi: 10.1186/s12885-019-6470-y (PMC6937680; doi:10.1186/s12885-019-6470-y)

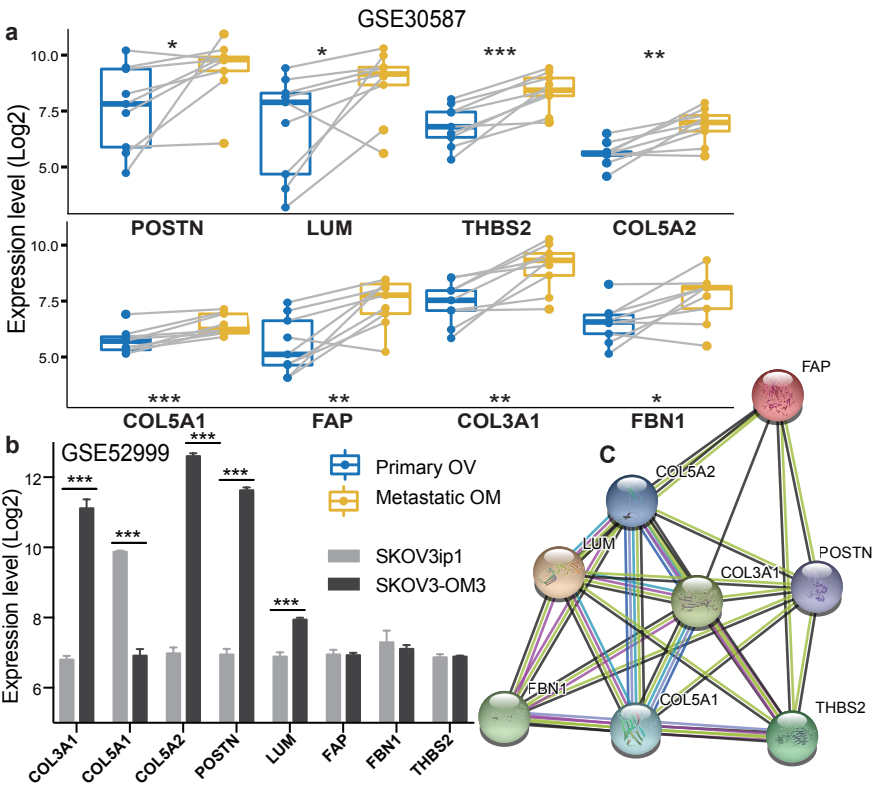

Supplement: Supplementary file 2 — Additional file 2: Figure. S1. (a) Paired t-test revealed that all eight genes were significantly elevated in omental metastases compared with the corresponding primary ovarian tumors in the dataset GSE30587. (b) Four genes (POSTN, LUM, COL3A1, COL5A2) of the LMGS were remarkably elevated, while COL5A1 was significantly down-regulated in SKOV3-OM3 (subpopulations derived from omental tumors in guest mice of the parabiosis models, representing omental metastases generated through a hematogenous route), compared to SKOV3ip1 intraperitoneal injected to the host mice (representing the primary tumors) in the dataset GSE52999. (c) Genes of the LMGS were likely to form a biologically functional network based on PPI analysis. Primary OV: primary ovarian cancer samples, Metastatic OM: omental metastases of ovarian cancer. * P < 0.05, ** P < 0.01, *** P < 0.001 [file 12885_2019_6470_MOESM2_ESM.pdf]

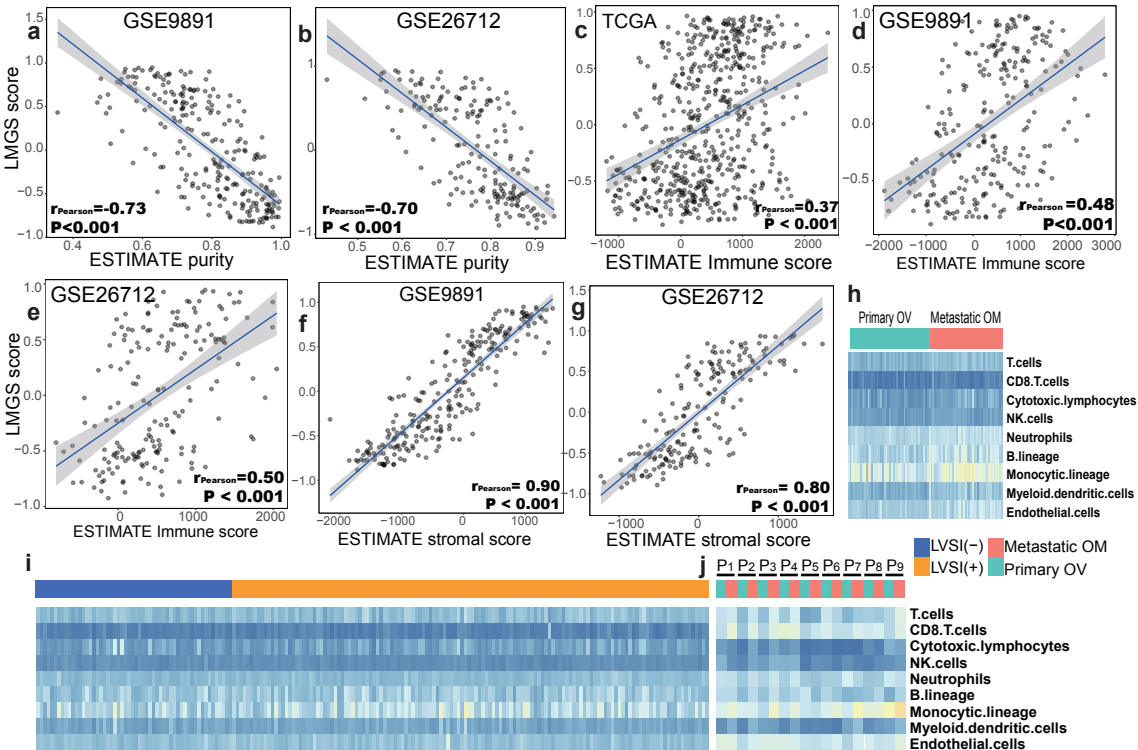

Supplement: Supplementary file 4 — Additional file 4: Figure. S3. The significant and negative correlation between the activation of the LMGS and tumor purity was validated in (a) GSE9891 and (b) GSE26712. (c-e) The positive correlation between the expression of the LMGS and immune cell infiltration was significant but relatively weak. The activation of the LMGS was positively correlated with mesenchymal infiltration in serous ovarian cancer samples from (f) GSE9891 and (g) GSE26712. The infiltration of immunocytes was similar between the primary ovarian cancer samples with LVSI-positive status versus LVSI-negative ones. A similar trend was observed in omental metastases compared with primary lesions in (h) dataset GSE2109 and was validated in (i) paired samples from dataset GSE30587 [file 12885_2019_6470_MOESM4_ESM.pdf]

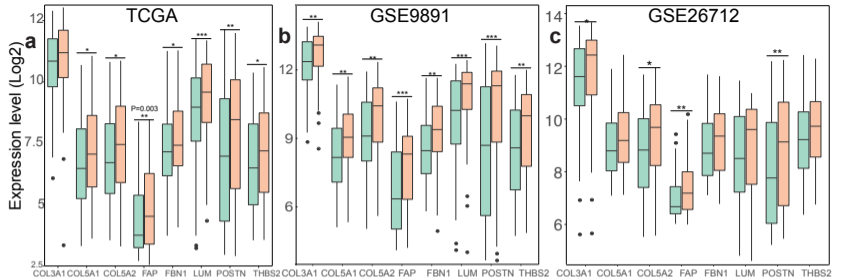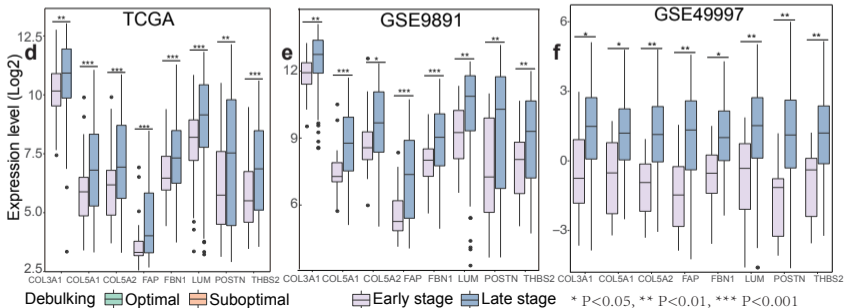

Supplement: Supplementary file 5 — Additional file 5: Figure. S4. Genes of the LMGS were remarkably elevated in (a-c) patients undergoing suboptimal cytoreduction and (d-f) those with late-stage serous ovarian cancer [file 12885_2019_6470_MOESM5_ESM.pdf]
