# Supplementary material for: Gene signature characteristic of elevated stromal infiltration and activation is associated with increased risk of hematogenous and lymphatic metastasis in serous ovarian cancer
Source: BMC Cancer. 2019 Dec 30;19:1266. doi: 10.1186/s12885-019-6470-y (PMC6937680; doi:10.1186/s12885-019-6470-y)
Supplement: Supplementary file 3 — Additional file 3: Figure. S2. All eight genes of the LMGS were highly enriched in the C1 molecular subtype of (a) the Tothill dataset and (b) in the TCGA mesenchymal subtype. (c) The expression levels of individual gene of the LMGS were significantly elevated in tumor stroma compared with the epithelial components in dataset GSE38666. A similar tendency was observed in paired samples in (d) dataset GSE115635, as well as in stromal components compared with normal ovarian stroma in (e) dataset GSE40595. [file 12885_2019_6470_MOESM3_ESM.pdf]

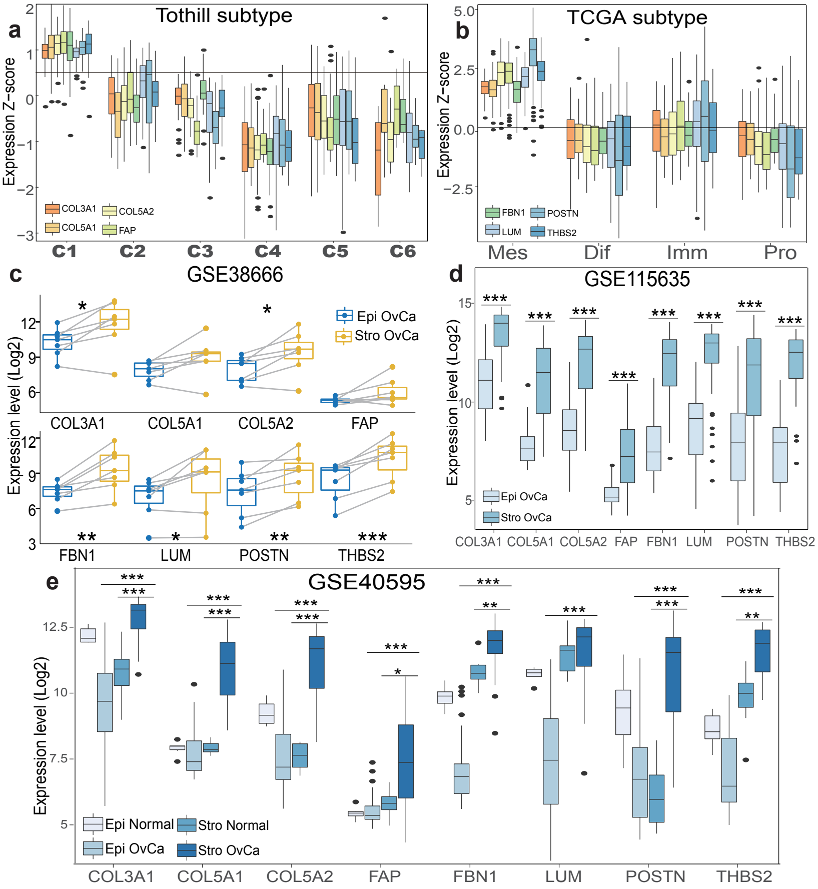

Mes: mesenchymal, Dif: differentiated, Imm: immunoreactive, Pro: proliferative
Epi/Stro OvCa: epithelial/stromal components in ovarian cancer samples
Epi/Stro normal: normal human ovarian epithelium/stroma samples
* P<0.05, ** P<0.01, *** P<0.001
